# Antibiogram, prevalence of methicillin-resistant and multi-drug resistant *Staphylococcus* spp. in different clinical samples

**DOI:** 10.1016/j.sjbs.2022.103432

**Published:** 2022-09-05

**Authors:** Ehssan H. Moglad, Hisham N. Altayb

**Affiliations:** a Department of Pharmaceutics, College of Pharmacy, Prince Sattam bin Abdulaziz University, P.O.Box 173, Alkharj 11942, Saudi Arabia; b Department of Microbiology, Medicinal and Aromatic Plants and Traditional Medicine Research Institute (MAPTMRI), P.O. Box 2404, National Center for Research, Khartoum, Sudan; c Department of Biochemistry, Faculty of Science, King Abdulaziz University, Building A 90, Jeddah 21589, Saudi Arabia

**Keywords:** Extensive drug-resistant, Antibiotics profile, βeta-lactamase, Multidrug-resistant, *S. aureus*, Khartoum-Sudan, MRSS, Methicillin-resistant *Staphylococcus* spp, MRSA, methicillin-resistant *S. aureus*, MDR, Multidrug-resistant, CLSI, Clinical and Laboratory Standards Institute, XRD, Extensively drug-resistant, PDR, Pandrug-resistant, AMR, Antimicrobial-resistant, ATCC, American Type Culture Collection

## Abstract

Methicillin-resistant *Staphylococcus* spp. (MRSS) are causing numerous forms of illness in humans ranging from mild to fatal infections. We need to investigate the resistant pattern for different clinical isolates to control the resistance phenomena. This study was designed to provide the resistance pattern of isolated *Staphylococcus* spp. from various clinical samples in Khartoum State and to elucidate the frequencies of Multidrug-resistant (MDR), Extensively drug-resistant (XDR) and pan-drug resistant (PDR). Two hundred and ten bacterial isolates were from different sources (catheter tip, sputum, vaginal swab, urine, tracheal aspirate, blood, pus, nasal swab, stool, throat swab, pleural fluid, and ear swab). Isolates were identified based on their morphological characters and biochemical reaction. Antibiotics susceptibility screening was performed using twenty-three antibiotics from eighteen classes against all isolated *Staphylococcus* spp. following the Clinical and Laboratory Standards Institute (CLSI) guideline. The result revealed that out of 63 Gram-positive isolated bacteria, 52 (82.5%) were *Staphylococcus* spp. with a high incidence of *S. aureus* 37(71.2%). Out of all *Staphylococcus* spp., 38 (73.1%) were Methicillin-resistant (MR). The prevalence of MDR was higher in *S. aureus* (89.2%) than in *S. epidermidis* (75%). All *Staphylococcus* spp. displayed resistance to ampicillin and penicillin, while all *S. aureus* were sensitive to daptomycin and fosfomycin. One isolate was XDR possible PDR, while no PDR was reported in all isolated bacteria. This study provided evidence for the antimicrobial-resistant (AMR) burden in Sudan and highlighted the need for a practical and functional stewardship program to reduce the unreasonable costs of antibiotics.

## 1 Introduction

*Staphylococcus* spp. is a bacterial pathogen that quickly obtains antibiotic resistance (Kot et al., 2020, Boucher et al., 2009). Antibiotics are one of our most powerful tools for preventing life-threatening infections, and bacteria become antibiotic-resistant by the capability to defeat the drugs used to kill them, which is a critical global public health challenge of our time. Now, people live in an era when people die because of untreatable infections due to the emergence and spread of antibiotic resistance (CDC, 2019). Moreover, microbial infections with antimicrobial-resistant strains make the disease worse by increasing the possibility of mortality and rising treatment expenses compared to disease by susceptible strains (Mulvey and Simor, 2009).

Furthermore, methicillin-resistant *S. aureus* (MRSA) is one of the significant problems globally which can cause both community and healthcare-acquired infections; it is reported that infections by MRSA affect more than 150,000 patients yearly in the healthcare setting alone in the European Union (Baldan et al., 2009, Köck et al., 2010), the emergence of resistant bacterial pathogens mainly due to extensive and prolonged use of antibiotics. It affects increasing morbidity and mortality rates (Tsige et al., 2020). The MRSA infection is multifold (Ben Zakour et al., 2008, Holmes et al., 2005) and is connected with worse outcomes, extended hospital stay, more treatment expenditure, and high mortality (Zahar et al., 2005, Shorr et al., 2006).

According to the WHO, nearly 80 % of *S. aureus* infections in Africa are resistant to methicillin, making the traditional antibiotics worthless for infection treatment. (WHO, 2014). Other studies in Ethiopia revealed that MRSA is a significant problem in public health (Kahsay et al., 2014, Godebo et al., 2013, Dilnessa and Bitew, 2016). In addition, in Sudan, one of the African countries with a border with Ethiopia, the prevention and governor policies are not well recognised to reduce MRSA. Antibiotics are routinely and incorrectly utilised, increasing the prevalence of drug resistance strain bacteria like MRSA. Therefore, exploring increased drug-resistant MRSA is essential for controlling and treating staphylococcal infections.

Accordingly, global epidemiological surveillance is critical to complete antimicrobial-resistant (AMR) response. Knowledge of local and provincial AMR is essential for medical selection formation. Nonetheless, there is a lack of investigation capabilities for AMR requests across Sudan, and current AMR records are sparse. According to our knowledge, no previous studies in the study area. Therefore, we aim to study the current prevalence of clinical isolates of *Staphylococcus spp*. and their resistance profile, including the determination of the MDR, XDR and PDR. In general, the findings of this study can be used as a source to develop a guide to reduce the burden of MRSA, and it provides modernised information that is useful for health care professionals responsible for patient supervision and monitoring the emergence of AMR.

## 2 Material and methods

### 2.1 Study population and sample size

This study was cross-sectional and conducted at three hospitals in Khartoum, Sudan (Omdurman, Soba and Bahri Teaching Hospitals). The study lasted six months, from October 2019 to March 2020. A total of 210 clinical bacterial isolates were recovered from the catheter tip, sputum, vaginal swab, urine, tracheal aspirate, blood, pus, nasal swab, stool, throat swab, pleural fluid, and ear swab samples. All specimens received in the hospital's microbiology laboratory were included during the study.

### 2.2 Culturing of bacterial isolate

According to the sample sources, the specimens were cultured in XLD, MacConkey Agar and Blood agar and incubated at 37 °C for 24 h. The sample showed dense pure colonies were examined for Gram stain (Cheesbourgh, 2006).

### 2.3 Identification of *Staphylococcus* spp.

All Gram-positive bacteria (GPB) were tested for catalase production, and positive bacteria were identified by culture characteristics on Mannitol Salt Agar (MSA) and blood agar. Biochemical reactions, such as DNase and coagulase production tests, are also used to recognise the species.

### 2.4 Detection of methicillin-resistant *Staphylococcus* spp. (MRS) by cefoxitin disc screen test

The susceptibility profile of *Staphylococcus* spp. to cefoxitin (30 μg) was carried out on Mueller-Hinton agar plates using the disc diffusion technique. The tested bacterial isolate was suspended on physiological saline, and then the turbidity was compared with a 0.5 McFarland standard. After incubation at 35 °C for 24 h, interpretation of inhibition zone performed using CLSI guidelines 2017 as fallows; *S. aureus*, resistant ≤ 21 mm and sensitive ≥ 22 mm whereas MRS were cefoxitin resistant. For quality control, *S. aureus* ATCC BAA 2313 was used for MRSA, and *S. aureus* ATCC BAA 1026 was used in each experiment. (CLSI, 2017).

### 2.5 Antimicrobial susceptibility profile of isolated *Staphylococcus* spp.

All *Staphylococcus* spp. isolates were investigated for antibiotic susceptibility using a disc diffusion test for common antibiotics used in the treatment of *Staphylococcus* spp infections, as following discs: azithromycin (15 μg), erythromycin (15 μg), ciprofloxacin (5 μg), moxifloxacin (5 μg), levofloxacin (5 μg), clindamycin (2 μg), linezolid (10 μg), rifampin (5 μg), teicoplanin (30 μg), mupirocin (5 μg), imipenem (10 μg), gentamicin (10 μg), ampicillin (10 μg), oxacillin (5 μg), penicillin (6 μg), amoxicillin-clavulanate (20 + 10 μg), fusidic acid (10 μg), trimethoprim/ sulfamethoxazole (1.25 + 23.75 μg), tetracycline (30 μg), daptomycin (30 μg), fosfomycin (50 μg), synercid (quinupristin and dalfopristin, 15 μg), and vancomycin (30 μg). The diameter of the inhibition zone was interpreted as resistant, intermediate and susceptible according to CLSI guidelines 2017. *S. aureus* ATCC 25,923 was used for quality control (CLSI, 2017).

### 2.6 Detection of MDR and XDR

MDR was defined as acquired resistance to at least one antimicrobial agent from 3 or more antimicrobial classes. XDR is characterised by resistance to at least one drug in all antibiotic types except two or fewer; bacterial isolates are only sensitive to one or two categories. The PDR is resistant to all groups of antimicrobial agents (Basak et al., 2016).

### 2.7 Statistical analysis

Statistical package for social sciences (SPSS) software programme version 20 (SPSS Inc., Chicago, IL) was used for analysing all data. Descriptive data is displayed as a percentage. A *P-value* of ≤ 0.05 was considered for significant association between sources of samples and isolated bacteria.

## 3 Results

### 3.1 Identification of samples from different sources

Out of two-hundred and ten isolates, 63 (30 %) were GPB, of this, *S. aureus* (58.7 %), *S. epidermidis* (12.7 %), *S. heamolyticus* (4.8 %), S*. cohnii* (1.6 %), *S. hominis* (1.6 %), *S. sciuri* (1.6 %), *S. lugdunensis* (1.6 %), *Enterococcus* spp. (6.4 %), *Streptococcus* spp. (11.2 %). Most of the isolated bacteria were from nasal swabs, pus, blood culture, and urine (Table 1).

**Table 1:** Prevalence and association between Gram-positive bacteria and sources of samples.

Gram-positive bacteria		**Source of samples**									**Total**
		**vaginal swab**	**urine**	**sputum**	**blood**	**tracheal aspirate**	**pus**	**throat swab**	**nasal swab**	**ear swab**	
*S. aureus*	N	1	2	2	1	3	13	0	15	0	37
	%	1.6	3.2	3.2	1.6	4.8	20.6	0.0	23.8	0.0	58.7
*S. epidermidis*	N	0	1	0	4	0	2	0	0	1	8
	%	0.0	1.6	0.0	6.3	0.0	3.2	0.0	0.0	1.6	12.7
*S. heamolyticus*	N	0	2	0	1	0	0	0	0	0	3
	%	0.0	3.2	0.0	1.6	0.0	0.0	0.0	0.0	0.0	4.8
*S. cohnii*	N	0	0	0	0	1	0	0	0	0	1
	%	0.0	0.0	0.0	0.0	1.6	0.0	0.0	0.0	0.0	1.6
*S. hominis*	N	0	0	0	1	0	0	0	0	0	1
	%	0.0	0.0	0.0	1.6	0.0	0.0	0.0	0.0	0.0	1.6
*S. sciuri*	N	0	0	0	0	0	1	0	0	0	1
	%	0.0	0.0	0.0	0.0	0.0	1.6	0.0	0.0	0.0	1.6
*S. lugdunensis*	N	0	0	0	0	0	1	0	0	0	1
	%	0.0	0.0	0.0	0.0	0.0	1.6	0.0	0.0	0.0	1.6
*Enterococcus* spp.	N %	0 0.0	3 4.8	0 0.0	1 1.6	0 0.0	0 0.0	0 0.0	0 0.0	0 0.0	4 6.4
*Streptococcus* spp.	N %	0 0.0	0 0.0	2 3.2	1 1.6	0 0.0	0 0.0	1 1.6	3 4.8	0 0.0 0.0	7 11.2
Total	N	1	8	4	9	4	17	1	18	1	63
	%	1.6	12.7	6.3	14.3	6.3	27.0	1.6	28.6	1.6	100.0
*P-value*	0.001										

Out of 63 GPB, *Staphylococcus* spp. was 52 (82.5 %) isolates as follows: *S. aureus* 37(71.2 %), *S. epidermidis* 8(15.4 %), *S. heamolyticus* 3(5.8 %), and 1(1.9 %) for *S. cohnii, S. hominis, S. sciuri* and *S. lugdunensis.*

### 3.2 Detection of methicillin-resistant *Staphylococcus* spp. (MRSS) by cefoxitin disc screen test

Of 52 isolated *Staphylococcus* spp., 38 (73.1 %) were positive for the Cefoxitin disc screen. The results are demonstrated in Table 2.

### 3.3 Detection of MDR, XDR and PDR

MDR was predominant. Of all isolated species, only *S. cohnii* are resistant to all tested antibiotics except for mupirocin which was not tested, so it is considered XDR and possible PDR. The results are displayed in Table 2.

**Table 2:** Prevalence of MDR and MRSS among isolated *Staphylococcus* spp.

Bacteria	Number and percentage of isolates		**MDR**		**MRSS (Cefoxitin test)**	
			**YES**	**NO**	**POS**	**NEG**
*S. aureus*	N	37	33	4	25	12
	%	100	89.2	10.8	67.6	32.4
*S. epidermidis*	N	8	6	2	7	1
	%	100	75	25	87.5	12.5
*S. heamolyticus*	N	3	3	0	3	0
	%	100	100	0	100	0
*S. cohnii*	N	1	1	0	1	0
	%	100	100	0	100	0
*S. hominins*	N	1	1	0	1	0
	%	100	100	0	100	0
*S. sciuri*	N	1	1	0	1	0
	%	100	100	0	100	0
*S. lugdunensis*	N	1	1	0	0	1
	%	100	100	0	0	100
*P-value*			0.001		0.001	

Key: MDR: multidrug-resistant bacteria, MRSS: Methicillin-resistant *Staphylococcus* spp.

### 3.4 Antimicrobial susceptibility profile of isolated *Staphylococcus* spp.

Twenty-three different antibiotics belong to eighteen different classes used in this study, and the result was interpreted according to CLSI guidelines. All *S. aureus* were sensitive to daptomycin and fosfomycin, and only two isolates were resistant to teicoplanin and rifampin. While all *Staphylococcus* spp. were resistant to penicillin and ampicillin, the result is shown in Table 3..

**Table 3:** Antimicrobial resistance pattern of *Staphylococcus* spp. isolated from different clinical samples.

Name		**CIP**	**LVX**	**MXF**	**AZM**	**E**	**DAP**	**AM**	**FOS**	**CM**	**FA**	**GM**	**IPM**
*S. aureus*	N	14	13	12	22	15	0	37	0	9	2	11	26
	%	37.8	35.1	32.4	59.5	40.5	0.0	100	0.0	24.3	5.4	29.7	70.3
*S. epidermidis*	N	4	4	2	5	5	0	8	1	3	1	3	7
	%	50	50	25	62.5	62.5	0.0	100	12.5	37.5	12.5	37.5	87.5
*S. heamolyticus*	N	1	1	0	1	1	0	3	0	1	0	1	3
	%	33.3	33.3	0.0	33.3	33.3	0.0	100	0.0	33.3	0.0	33.3	100
*S. cohnii*	N	1	1	1	1	1	1	1	1	1	1	1	1
	%	100	100	100	100	100	100	100	100	100	100	100	100
*S. hominis*	N	0	0	0	1	1	0	1	0	0	0	0	1
	%	0.0	0.0	0.0	100	100	0.0	100	0.0	0.0	0.0	0.0	100
*S. sciuri*	N	0	0	0	0	0	0	1	0	1	0	1	1
	%	0.0	0.0	0.0	0.0	0.0	0.0	100	0.0	100	0.0	100	100
*S. lugdunensis*	N	0	0	0	1	0	0	1	0	0	0	0	0
	%	0.0	0.0	0.0	100	0.0	0.0	100	0.0	0.0	0.0	0.0	0.0

Name		**P**	**OX**	**VA**	**TEC**	**SYN**	**NEXT**	**TE**	**RA**	**LZD**	**AMC**	**MAP**
*S. aureus*	N	37	25	3	2	3	3	7	2	16	26	13
	%	100	67.6	8.1	5.4	8.1	8.1	18.9	5.4	43.2	70.2	35.1
*S. epidermidis*	N	8	7	0	0	0	2	2	0	1	7	2
	%	100	87.5	0.0	0.0	0.0	25	25	0.0	12.5	87.5	25
*S. heamolyticus*	N	3	3	0	0	0	0	2	0	0	3	0
	%	100	100	0.0	0.0	0.0	0.0	66.7	0.0	0.0	100	0.0
*S. cohnii*	N	1	1	1	1	1	1	1	1	1	1	NT
	%	100	100	100	100	100	100	100	100	100	100	
*S. hominis*	N	1	1	0	0	0	0	0	0	0	1	0
	%	100	100	0.0	0.0	0.0	0.0	0.0	0.0	0.0	100	0.0
*S. sciuri*	N	1	1	0	0	1	0	1	1	0	1	NT
	%	100	100	0.0	0.0	100	0.0	100	100	0.0	100	
*S. lugdunensis*	N	1	0	0	0	0	0	0	0	1	0	1
	%	100	0.0	0.0	0.0	0.0	0.0	0.0	0.0	100	0.0	100

Key: N: Number of resistant bacteria, CIP: Ciprofloxacin, MXF: Moxifloxacin, LVX: Levofloxacin, AZM: Azithromycin, E: Erythromycin, DAP: Daptomycin, AM: Ampicillin, FOS: Fosfomycin, CM: Clindamycin, FA: Fusic acid, GM: Gentamicin, IPM: Imipenem.

P = Pencillin, OX = Oxacillin, VA = Vancomycin, TEC = Teicoplanin, SYN = Synercid, SXT = Trimethoprim-sulfamethoxazole, TE = Tetracycline, RA = Rifampin, LZD = Linezolid, AMC = Amoxicillin-clavulanate, MUP = Mupirocin, NT = not tested.

## 4 Discussion

In this study, we found the most common isolated species is *S. aureus* 37(71.2 %)*,* followed by *S. epidermidis* 8(15.4 %); this might be because *S. aureus* is more virulent and *S. epidermidis* is an opportunistic pathogen (Otto, 2009). MRSA prevalence was 67.6 %, and this finding is the highest one when comparing it between African and non-African countries; such as the MRSA reported in Kenya (53.4 %) (Wangai et al., 2019), Uganda (41 %) (Ojulong et al., 2010), Addis Ababa (13.2 %) (Tsige et al., 2020), Eretria (9 %) (Naik and Teclu, 2009), Cameroon (13.16 %) (Bissong et al., 2016), Nigeria (5.8 %) (Ghebremedhin et al., 2009), Tanzania (4.3 %) (Mshana et al., 1970) and Brazil (5.6 %) (Almeida et al., 2014). This high frequency of MDR in our study could be related to a high level of antibiotic use, which could be due to accessibility or low purchase prices of drugs.

In this study, all *Staphylococcus* spp. including *S. aureus* was resistant to penicillin and ampicillin. This finding is unlike the results obtained from Tanzania (97 %) (Mshana et al., 1970), Nigeria (95.8 %) (Uwaezuoke and Aririatu, 2004), and ultimately agreed with a study done in Ethiopia (East border country) which found that all MRSA were 100 % resistant to penicillin and ampicillin (Kejela and Bacha, 2013).

Interestingly, All *S. aureus* were sensitive to daptomycin and fosfomycin, and numerous proportions of *S. aureus* isolates, including MRSA, were susceptible to vancomycin, synercid, and trimethoprim-sulfamethoxazole were (91.9 %), fusic acid, teicoplanin and rifampin were (94.6 %). These results agree with a study that reported that the susceptibility of *S. aureus* to vancomycin was 91.7 % (DeLeo et al., 2010). In a review of similar work, these findings disagreed with the study done in Southwest Ethiopia reported that 97 % of all *S. aureus* isolates were susceptible to vancomycin, 94.7 % to trimethoprim-sulfamethoxazole (Kejela and Bacha, 2013), other study said 96.9 % of *S. aureus* isolates were susceptible to vancomycin (Rijal et al., 2008).

MRSA percentage among all *S. aureus* isolates was 67.6 %, higher than the previous study done in Sudan, which was 44.6 % (Elboshra et al., 2020). While in Italy reached 35.8 % (Campanile et al., 2015), above 40 % in Philippines (Juayang et al., 2014), and in Saudi Arabia 64.6 % (Moglad, 2021). This result is alarming because MRSA has limited drug choices to treat infections caused by them.

In our study, the first study to screen Twenty-three antibiotics belonging to eighteen different classes of antibiotics, and a significant percentage (88.5 %) of *Staphylococcus* spp. isolates were MDR. The resistance pattern ranged from five antibiotics to fifteen antibiotics, and for *S. aureus* MDR was 89.2 % which is higher than that reported in Italy 35.8 % (Campanile et al., 2015) and Ethiopia (82.3 %) (Kahsay et al., 2014). Many of our MDR isolates were MRSA, which shows that the opportunities to treat disease caused by MDR MRSA with conventional antibiotics are very narrow.

Unfortunately, our results showed that *S. cohnii* was resistant to all tested antibiotics, with an exception for mupirocin was not tested, which made it XDR and possible PDR. Alarmingly resistant bacteria could share their resistance genes even with bacteria not exposed to antibiotics (CDC, 2019). As previously reported in the literature, *S. aureus* develops resistance to several antibiotics by gaining elements by horizontal transferring of mobile genetic material, modifying the drug-binding sites on molecular targets by mutations, and expressing endogenous efflux pumps (Foster, 2017). MRSA is an important human pathogen with growing resistance to presently used antimicrobial remedies (Pokharel et al., 2019).

## 5 Conclusion

This study focused on the current scenario of antimicrobial-resistant *Staphylococcus* spp. and highlighted the frequencies of MDR, XDR, and PDR among clinical isolates of *Staphylococcus* spp. in Khartoum state Sudan and the incidence of MDR *Staphylococcus* spp. was high.

Also, this study showed the current resistant patterns, which are essential for the active treatment of diseases caused by MDR and MRSA. This study might evidence the serious need for monitoring and dealing with the development of MDR strain. Finally, this study recommended that antibiotic use be improved, reduce unnecessary uses, and ensure improved access to antibiotics in Sudan.

## Declaration of Competing Interest

The authors declare that they have no known competing financial interests or personal relationships that could have appeared to influence the work reported in this paper

## References

[b0005] Almeida G.C., dos Santos M.M., Lima N.G., Cidral T.A., Melo M.C., Lima K.C. (2014). Prevalence and factors associated with wound colonisation by Staphylococcus spp. and Staphylococcus aureus in hospitalised patients in inland northeastern Brazil: a cross-sectional study. BMC Infect. Dis..

[b0010] Baldan R., Tassan Din C., Semeraro G., Costa C., Cichero P., Scarpellini P., Moro M., Cirillo D.M. (2009). Severe community-onset infections in healthy individuals caused by community-acquired MRSA in an Italian teaching hospital, 2006–2008. J. Hosp. Infect..

[b0015] Basak S., Singh P., Rajurkar M. (2016). Multidrug resistant and extensively drug resistant bacteria: a study. J. Pathog..

[b0185] BEN ZAKOUR, N. L., GUINANE, C. M. & FITZGERALD, J. R. 2008. Pathogenomics of the staphylococci: insights into niche adaptation and the emergence of new virulent strains. *FEMS Microbiology Letters,* 289**,** 1-12. 10.1111/j.1574-6968.2008.01384.x 19054087

[b0020] Bissong M., Wirgham T., Enekegbe M., Niba P., Foka F. (2016). Prevalence and antibiotic susceptibility patterns of methicillin resistant staphylococcus aureus in patients attending the Laquintinie Hospital Douala, Cameroon. *Eur. J. Clin. Biomed. Sci.*.

[b0025] Boucher H.W., Talbot G.H., Bradley J.S., Edwards J.E., Gilbert D., Rice L.B., Scheld M., Spellberg B., Bartlett J. (2009). Bad bugs, no drugs: no ESKAPE! an update from the Infectious Diseases Society of America. Clin. Infect. Dis..

[b0030] Campanile F., Bongiorno D., Perez M., Mongelli G., Sessa L., Benvenuto S., Gona F., Varaldo P.E., Stefani S. (2015). Epidemiology of Staphylococcus aureus in Italy: First nationwide survey, 2012. J. Glob. Antimicrob. Resist..

[b0035] CDC 2019. Antibiotic Resistance Threats in the United States. *Atlanta, GA: U.S. Department of Health and Human Services, CDC*.

[b0040] CHEESBOURGH, M. 2006. Medical laboratory manual for tropical countries. 2nd ed. *England: Butterworth-Heineman Ltd*.

[b0045] CLSI 2017. Pereferance standard for antimicrobial susceptability testing. *CLSI suplement M 100,* 27th edu.

[b0050] Deleo F.R., Otto M., Kreiswirth B.N., Chambers H.F. (2010). Community-associated meticillin-resistant Staphylococcus aureus. Lancet.

[b0055] Dilnessa T., Bitew A. (2016). Prevalence and antimicrobial susceptibility pattern of methicillin-resistant Staphylococcus aureus isolated from clinical samples at Yekatit 12 Hospital Medical College, Addis Ababa, Ethiopia. *BMC Infect. Dis.*.

[b0060] Elboshra M.M.E., Hamedelnil Y.F., Moglad E.H., Altayb H.N. (2020). Prevalence and characterisation of virulence genes among methicillin-resistant Staphylococcus aureus isolated from Sudanese patients in Khartoum state. New Microb. New Infect..

[b0065] FOSTER, T. J. 2017. Antibiotic resistance in Staphylococcus aureus. Current status and future prospects. *FEMS Microbiol Rev,* 41**,** 430-449. 10.1093/femsre/fux007 28419231

[b0070] Ghebremedhin B., Olugbosi M.O., Raji A.M., Layer F., Bakare R.A., König B., König W. (2009). Emergence of a community-associated methicillin-resistant Staphylococcus aureus strain with a unique resistance profile in Southwest Nigeria. J. Clin. Microbiol..

[b0075] Godebo G., Kibru G., Tassew H. (2013). Multidrug-resistant bacterial isolates in infected wounds at Jimma University Specialized Hospital, Ethiopia. Ann. Clin. Microbiol. Antimicrob..

[b0080] Holmes A., Ganner M., McGuane S., Pitt T.L., Cookson B.D., Kearns A.M. (2005). Staphylococcus aureus isolates carrying Panton-Valentine leucocidin genes in England and Wales: frequency, characterisation, and association with clinical disease. J. Clin. Microbiol..

[b0085] JUAYANG, A. C., DE LOS REYES, G. B., DE LA RAMA, A. J. & GALLEGA, C. T. 2014. Antibiotic Resistance Profiling of Staphylococcus aureus Isolated from Clinical Specimens in a Tertiary Hospital from 2010 to 2012. *Interdiscip Perspect Infect Dis,* 2014**,** 898457. 10.1155/2014/898457 PMC4167206 25258625

[b0090] Kahsay A., Mihret A., Abebe T., Andualem T. (2014). Isolation and antimicrobial susceptibility pattern of Staphylococcus aureus in patients with surgical site infection at Debre Markos Referral Hospital, Amhara Region, Ethiopia. *Arch. Public Health*.

[b0095] Kejela T., Bacha K. (2013). Prevalence and antibiotic susceptibility pattern of methicillin-resistant Staphylococcus aureus (MRSA) among primary school children and prisoners in Jimma Town, Southwest Ethiopia. Ann. Clin. Microbiol. Antimicrob..

[b0100] KÖCK, R., BECKER, K., COOKSON, B., VAN GEMERT-PIJNEN, J. E., HARBARTH, S., KLUYTMANS, J., MIELKE, M., PETERS, G., SKOV, R. L., STRUELENS, M. J., TACCONELLI, E., NAVARRO TORNÉ, A., WITTE, W. & FRIEDRICH, A. W. 2010. Methicillin-resistant Staphylococcus aureus (MRSA): burden of disease and control challenges in Europe. *Euro Surveill,* 15**,** 19688. 10.2807/ese.15.41.19688-en 20961515

[b0105] Kot B., Wierzchowska K., Piechota M., Grużewska A. (2020). Antimicrobial resistance patterns in methicillin-resistant Staphylococcus aureus from patients hospitalized during 2015–2017 in hospitals in Poland. Med. Princip. Pract..

[b0110] MOGLAD, E. H. 2021. Loranthus acaciae: Alternative medicine for β-lactamase producer and methicillin-resistant Staphylococcus aureus. *Saudi J Biol Sci,* 28**,** 1835-1839. 10.1016/j.sjbs.2020.12.029 PMC7938108 33732069

[b0115] Mshana S.E., Kamugisha E., Miramb M., Chalya P., Rambau P., Mahalu W., Lyamuya E. (1970). Prevalence of clindamycin inducible resistance among methicillin-resistant Staphylococcus aureus at Bugando Medical Centre, Mwanza, Tanzania. Tanzania J. Health Res..

[b0120] Mulvey M.R., Simor A.E. (2009). Antimicrobial resistance in hospitals: how concerned should we be?. CMAJ.

[b0125] Naik D., Teclu A. (2009). A study on antimicrobial susceptibility pattern in clinical isolates of Staphylococcus aureus in Eritrea. Pan Afr. Med. J..

[b0135] Ojulong J., Mwambu T., Jolobo M., Agwu E., Bwanga F., Najjuka C. (2010). Prevalence of methicillin-resistant staphylococcus aureus (MRSA) among isolates from surgical site infections in Mulago hospital-Kampala, Uganda. Internet J. Infect. Dis..

[b0140] OTTO, M. 2009. Staphylococcus epidermidis--the 'accidental' pathogen. *Nature reviews. Microbiology,* 7**,** 555-567. 10.1038/nrmicro2182 PMC2807625 19609257

[b0145] POKHAREL, S., RAUT, S. & ADHIKARI, B. 2019. Tackling antimicrobial resistance in low-income and middle-income countries. 4**,** e002104 10.1136/bmjgh-2019-002104 PMC6861125 31799007

[b0150] Rijal K.R., Pahari N., Shrestha B.K., Nepal A.K., Paudel B., Mahato P., Skalko-Basnet N. (2008). Prevalence of methicillin-resistant Staphylococcus aureus in school children of Pokhara. Nepal Med. Coll. J..

[b0155] Shorr A.F., Tabak Y.P., Gupta V., Johannes R.S., Liu L.Z., Kollef M.H. (2006). Morbidity and cost burden of methicillin-resistant Staphylococcus aureus in early onset ventilator-associated pneumonia. Crit. Care.

[b0160] TSIGE, Y., TADESSE, S., G/EYESUS, T., TEFERA, M. M., AMSALU, A., MENBERU, M. A. & GELAW, B. 2020. Prevalence of Methicillin-Resistant <i>Staphylococcus aureus</i> and Associated Risk Factors among Patients with Wound Infection at Referral Hospital, Northeast Ethiopia. *Journal of Pathogens,* 2020**,** 3168325. 10.1155/2020/3168325 PMC7271240 32566311

[b0165] Uwaezuoke J., Aririatu L. (2004). A survey of antibiotic-resistant Staphylococcus aureus strains from clinical sources in Owerri. J. Appl. Sci. Environ. Manage..

[b0170] Wangai F.K., Masika M.M., Maritim M.C., Seaton R.A. (2019). Methicillin-resistant Staphylococcus aureus (MRSA) in East Africa: red alert or red herring?. BMC Infect. Dis..

[b0175] WHO 2014. WHO’s First Global Report on Antibiotic Resistance, News Release. Geneva, Switzerland.

[b0180] ZAHAR, J. R., CLEC'H, C., TAFFLET, M., GARROUSTE-ORGEAS, M., JAMALI, S., MOURVILLIER, B., DE LASSENCE, A., DESCORPS-DECLERE, A., ADRIE, C., COSTA DE BEAUREGARD, M. A., AZOULAY, E., SCHWEBEL, C. & TIMSIT, J. F. 2005. Is methicillin resistance associated with a worse prognosis in Staphylococcus aureus ventilator-associated pneumonia? *Clin Infect Dis,* 41**,** 1224-31 10.1086/496923 16206094

